# Sauna bathing: a warm heart proves beneficial

**DOI:** 10.1007/s12471-015-0676-7

**Published:** 2015-04-01

**Authors:** E.E. van der Wall

**Affiliations:** Netherlands Society of Cardiology/Holland Heart House, Moreelsepark 1, 3511 EP Utrecht, The Netherlands

Commonly, cardiologists have concerns about exposing heart patients to the heat present in a sauna. In particular, sauna bathing leads to a significant increase in heart rate and reduction in total vascular resistance, thereby decreasing blood pressure. Over the years, it has been shown that sauna baths are well tolerated and pose no risk to healthy people from infancy to old age, including healthy women with uncomplicated pregnancy [1, 2]. In addition, there is a positive effect of sauna on lipid profile both in young, physically active, male subjects, and in young women [3, 4].

For patients with cardiovascular pathologies, it is still considered by many physicians (and thus their patients) that they should avoid sauna bathing. In 2008, mortality in saunas was described in a Swedish study performed between 1993 and 2002; it turned out that in 77 casualties, 34 (44 %) of these deaths were related to alcohol and 18 (23 %) to cardiovascular diseases [5]. In the same year, a Finnish study showed that the annual rate of death occurring while in a sauna was less than 2 per 100,000 inhabitants. Almost half (51 %) of the cases were natural deaths and exposure to heat was the cause of death in 25 %. Overall, 50 % of all cases were under the influence of alcohol. The main conclusion was that death in the sauna is a rare event, even in Finland where the frequency of sauna bathing is high [6]. Fortunately, more recent studies have shown that cardiovascular patients with essential hypertension, coronary heart disease, or postmyocardial infarction, who are stable and relatively asymptomatic in their everyday life, may take sauna baths without undue risk [7–10]. It has been documented that Waon therapy (repeated low-temperature sauna visits) improves myocardial ischemia due to chronic total occlusion of a coronary artery in association with improvement of vascular endothelial function [7]. For patients with congestive heart failure, sauna bathing under moderate and supervised conditions appears to be well tolerated and may be safe [8]. Repeated sauna therapy in patients with chronic heart failure improves exercise tolerance together with improvement in endothelial function [9] and reduced occurrence of ventricular arrhythmias [10]. As a rule of thumb, if a person can walk into a sauna, he or she can walk out of it [1].

Recent research published in the February 2015 issue of JAMA Internal Medicine evaluated the cardiovascular effects of sauna bathing by performing a large prospective study in male sauna bathers [11]. Generally, the study showed that long, hot sauna baths were associated with apparent health benefits, including fewer deaths from heart attacks, strokes, various heart-related conditions, and other causes. The Finnish group headed by Laukkanen [11] investigated the association of frequency and duration of sauna bathing with the risk of sudden cardiac death, fatal coronary heart disease, fatal cardiovascular disease, and all-cause mortality. The authors performed a prospective cohort study (Finnish Kuopio Ischemic Heart Disease Risk Factor Study) of a population-based sample of 2315 middle-aged (age 42–60 years) men from eastern Finland. Baseline examinations were conducted from 1 March 1984–31 December 1989. The investigators followed the participants until 2011.

More specifically, it was shown that men who enjoyed a sauna two or three times a week had a 23 % lower risk of experiencing a fatal episode of coronary heart disease or cardiovascular disease, compared with those who took just one sauna a week. These apparent health benefits for men who used the sauna four to seven times a week was even greater: they had a 48 % lower risk of similar incidents when compared with men who used the sauna only once a week. The final conclusion was that increased frequency of sauna bathing is associated with a reduced risk of sudden cardiac death, fatal coronary heart disease, fatal cardiovascular disease, and all-cause mortality, although the authors remark that further randomized studies are warranted to establish the potential mechanism that connects sauna bathing with cardiovascular health. It is also unfortunate that women were not included but, based on earlier sauna studies in women, similar effects might be anticipated. In an accompanying editorial comment, Rita Redberg (University of California, San Francisco, Editor-in-chief of JAMA Internal Medicine) stated, “*Although we do not know why the men who took saunas more frequently had greater longevity—whether it is the time spent in the hot room, the relaxation time, the leisure of a life that allows for more relaxation time or the camaraderie of the sauna—clearly time spent in the sauna is time well spent*” [12].

These findings could cause cardiologists to reconsider commonly held concerns about exposing heart patients to the heat present in a sauna. Apart from physical and mental well-being, patients might live longer when they regularly use a sauna bath. To paraphrase the rule of thumb, if a person can walk into a sauna, he or she can walk out of it but with a warmer and healthier heart.
